# Internet Survey Evaluation of Iliopsoas Injury in Dogs Participating in Agility Competitions

**DOI:** 10.3389/fvets.2022.930450

**Published:** 2022-07-08

**Authors:** Lindsey M. Fry, Nina R. Kieves, Abigail B. Shoben, Jessica K. Rychel, Arielle Pechette Markley

**Affiliations:** ^1^Red Sage Integrative Veterinary Partners Rehabilitation Clinic, Fort Collins, CO, United States; ^2^Department of Veterinary Clinical Sciences, College of Veterinary Medicine, The Ohio State University, Columbus, OH, United States; ^3^College of Public Health, Division of Biostatistics, The Ohio State University, Columbus, OH, United States; ^4^Veterinary Medical Center, The Ohio State University, Columbus, OH, United States

**Keywords:** agility, dog, iliopsoas, injury, muscle injuries, sports medicine, canine

## Abstract

**Objective:**

To describe risk factors associated with demographics, training, and competition for iliopsoas injury in dogs participating in agility competitions, as well as describe owner reported treatment and return to sport following injury.

**Procedures:**

An internet-based survey of agility handlers collected risk factor data for dogs participating in agility. Owners were asked questions about demographics, training, and competition as well as injury treatment and recovery if applicable. Associations between variables of interest and iliopsoas injury were estimated with logistic regression. The final risk factor model was built via modified backward selection, with all variables in the final model showing significant associations at *p* < 0.05.

**Results:**

Of the 4,197 dogs in the sample, 327 (7.8%) reported iliopsoas injury. The final model identified six risk factors for iliopsoas injury. A higher risk of iliopsoas injury was observed for the Border Collie breed, dogs with handlers who are veterinary assistants, dogs competing on dirt, dogs competing on artificial turf 6+ times a year, and dogs that trained with the 2 × 2 method for weave poles. Dogs that were not acquired with agility in mind were observed to have a decreased risk of injury. Factors like number of competition days and jump height were not significantly associated with risk of iliopsoas injury. Owners sought veterinary care for 88% of dogs with iliopsoas injury, including specialty care for 63%. Treatment most often included rest, home rehabilitation, formal rehabilitation, and/or oral medications. Most dogs (80%) were able to return to sport within 6 months, while 20% were out for longer than 6 months, or retired.

**Conclusion and Clinical Relevance:**

Iliopsoas injury can necessitate a significant amount of time off from training and competition, and even lead to retirement of dogs competing in agility. Some of the risk factors identified in this study can inherently not be modified (breed, intended use, and handler profession), but can be taken into consideration for injury prevention strategies. Competition and training risk factors that can be modified, such as weave training, may help to inform guidelines for best practices in management of the agility athlete.

## Introduction

Agility is one of the most popular international sporting activities for dogs and comes with an inherent risk for injury. Soft tissue injuries including strains, sprains, and contusions are commonly reported in agility dogs (1, 2). A recent, large-scale survey of injuries in agility dogs, found iliopsoas injuries to be the second most commonly reported injury (3). Dogs from the general population are also at risk of iliopsoas injury, with one study reporting that dogs presenting to an orthopedic service for hind limb muscle injuries were most frequently diagnosed with iliopsoas injury (4). Iliopsoas injury can result in extended absence from training and competition (5). Despite the frequency of muscle and tendon injuries seen in dogs, particularly iliopsoas injury, overall investigation in the veterinary literature is limited especially when compared to equine or human sports medicine. Recent studies have examined specific injuries, such as those involving the digits and cranial cruciate ligament, and offer more specific risk factors and potential modifications to athlete management, but none have focused specifically on iliopsoas injury (6, 7). Identifying risk factors for development of iliopsoas injury is important for advancing the areas of prevention, diagnosis, and treatment in order to improve welfare of our canine athletes.

The iliopsoas muscle is formed by the psoas and iliacus muscles and acts as an important flexor and stabilizer of the hip and vertebral column (8). The iliopsoas is prone to acute injury and strain when there is stretch while in eccentric contraction, which is common with a slip or fall, mis-jumping, or quick changes in direction (5, 8, 9). As with any muscle injury, if left undiagnosed or untreated these initial injuries can progress to become chronic in nature. Chronic iliopsoas injury is now more commonly recognized in canine athletes, and is suspected to be a result of repetitive microtraumas to the muscle secondary to altered gait mechanics (5, 8). Both acute and chronic injury can contribute to pathologic changes in muscle anatomy and physiology, evident via musculoskeletal ultrasound (5). Agility dogs diagnosed with iliopsoas injury commonly have decreased performance, reluctance to jump and lameness that is exacerbated by activity (9). On physical exam, these patients often have pain with direct palpation of the muscle belly or at the site of insertion on the lesser trochanter of the femur, and some patients have pain that is exacerbated with extension and internal rotation of the hip (5, 9). Pain is related to the primary muscle injury, and can also involve nerves or other surrounding soft tissues (5). The femoral nerve is at risk with iliopsoas injury, as it passes directly through the muscle belly of the psoas major muscle or between psoas major and iliacus muscle groups (10, 11).

Risk factors for muscular injury in both equine and human athletes are well defined and extensively studied. Determining risk factors for injury in canine agility athletes remains in its infancy, but is imperative in determining injury prevention strategies. Therefore, the primary aim of this study was to determine risk factors for iliopsoas injury in three categories: demographics, training, and competition. A secondary aim was to collect initial data on how agility dogs with iliopsoas injury were managed and how long it took for them to return to competition. We hypothesized competing more frequently and doing more runs per day would increase risk of iliopsoas injury. We also hypothesized that earlier full height jump training and full height obstacle training would increase risk of injury.

## Methods

Data from a previously described internet survey were utilized (3, 12, 13). Briefly, individuals were eligible if they had at least one dog who had competed in dog agility in the past 3 years. All owners were asked a variety of questions about demographics (both dog and handler), training factors (such as age starting training various obstacles), and competition factors (such as primary organization and details of typical trial weekends). Dogs that had ever had an iliopsoas injury that kept them from participating in agility for over a week were classified as having a history of iliopsoas injury. Follow-up questions were asked about the injury (or most significant injury if the owner reported more than one iliopsoas injury), including whether veterinary care was sought, who determined treatment, general therapeutic categories utilized, and return to competition timeframe. Specifics regarding how the injury was diagnosed, rehabilitation plans, and medication use were not asked.

Descriptive statistics (number, percent) were used to characterize treatments reported. For associations between variables of interest and iliopsoas injury, logistic regression was used with iliopsoas injury history as the outcome and variables of interest as predictors. All models were adjusted for dog age to account for differences in exposure time for injury history. Variables of interest were grouped into three blocks: demographic factors, competition factors, and training factors (12). Model building was conducted in three steps. In step 1, all variables were assessed for a possible association (*p* < 0.20) with iliopsoas injury in age-only adjusted models. Next, within each block, variables meeting criteria from step 1 were included in an initial model and then backward selection was done until all variables in the model showed some evidence of possible association at *p* < 0.20 (step 2). In step 3, all variables retained from the three models in step 2 were included in a final backward selection process until all remaining variables were significant at *p* < 0.05. We used an available case approach to missing data (after restricting to our primary sample) and analyses were conducted in Stata version 15 (3).

## Results

The sample of 4,197 dogs has been described previously (3, 13). Iliopsoas injury was reported by handlers for 327 (7.8%) dogs with strain the most common injury reported (12). Among those with strains (*n* = 281), 181 (64%) reported only one strain injury, while 69 (25%) reported two, and 31 (11%) reported three or more strain injuries. Owners reported seeking veterinary care in 288 (88%) of 326 cases (treatment information was missing on one), and 207 (63%) sought care from a veterinary specialist. Owners reported that treatment was predominantly determined by a veterinarian (*n* = 180, 55%), or a non-veterinary practitioner such as a chiropractor or massage therapist (*n* = 137, 42%), with the remainder reporting that treatment was determined by themselves or a member of their household (*n* = 8, 2%) or an agility friend (*n* = 1, 0.3%). Nearly all owners reported rest as part of the treatment plan (*n* = 300, 92%), with a substantial number also reporting at home rehabilitation exercises (*n* = 226, 69%), formal rehabilitation (*n* = 209, 64%), and medication use (*n* = 155, 48%).

**Figure 1:**
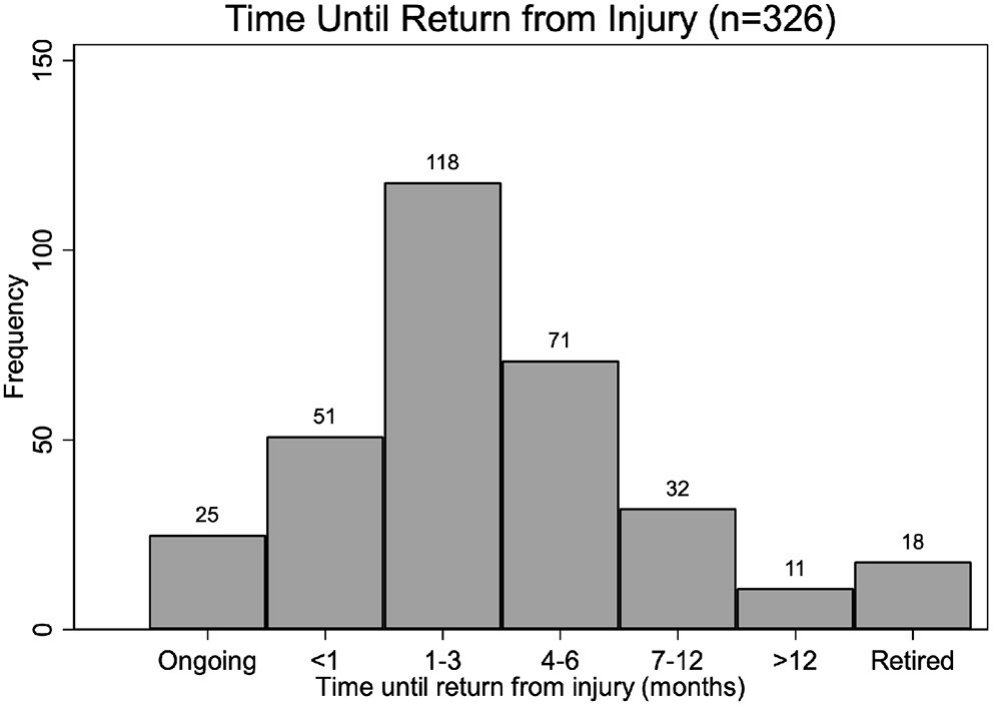
Reported time to return to agility training and competition from iliopsoas injury among 326 dogs.

Injury resolution information was available for 301 dogs (25 reported dogs actively undergoing treatment for iliopsoas injury at the time of the survey, 1 was missing). A majority of dogs (*n* = 169, 56%) were able to return to competition within 3 months, and 71 (24%) were able to return within 3–6 months. Forty-three dogs (14%) returned to competition after longer than 6 months, and 18 dogs (6%) were officially retired (Figure 1).

Many candidate variables showed some evidence of association with iliopsoas injury in age-only adjusted models (Table 1, Supplementary Tables 1–3). After model building, six factors remained in the final model (Table 2). Breed was strongly associated with iliopsoas injury, with Border Collies most likely to report an injury (OR: 1.99, 95% CI: 1.51 to 2.63). Dogs that were not acquired with agility in mind were less likely to report an iliopsoas injury (OR: 0.57, 95% CI: 0.41 to 0.78) than dogs that were acquired with agility in mind. Dogs of handlers who are veterinary assistants were more likely to report an iliopsoas injury, with relatively similar odds among the other categories of owners with and without veterinary medical training. Frequency of competing on turf and dirt were also both associated with risk of iliopsoas injury. Dogs that competed on artificial turf 6 or more times per year were more likely to report an iliopsoas injury (OR: 1.77, 95% CI: 1.32 to 2.37) compared to dogs who never competed on turf. Dogs who had ever competed on dirt were more likely to report an injury compared to dogs with no history on that surface (ORs 1.53 and 1.46 for 1–5 times per year and 6 or more times per year, respectively). Weave training method was also associated with iliopsoas injury; dogs who learned weaves by the 2 × 2 training method were more likely to report iliopsoas injury history. All other methods of training reported lower risk (ORs between 0.59 and 0.79), with the channel method associated with the lowest risk of injury.

**Table 1:** All factors considered in model building.

	***p <*** **0.2 in age-adjusted models (step 1)**	***p <*** **0.2 in block model building** **(step 2)**	**Retained in final model (step 3)**
**Demographic factors**			
Height & weight together	✓		
Breed	✓	✓	✓
Country/region	✓	✓	
Age brought dog home	✓		
How acquired (breeder, rescue, other)	✓		
Acquired with agility in mind	✓	✓	✓
Agility main sport focus	✓	✓	
Sex/neuter status			
Front dew claws			
Rear dew claws			
Docked tail			
Growth plate x-rays done	✓	✓	
Handler current age			
Handler gender	✓	✓	
Handler education			
Handler profession	✓		
Handler medical training	✓	✓	✓
Handler agility experience	✓		
Handler competed national level	✓		
Handler competed international level			
**Competition factors**			
Primary organization	✓		
Dog highest level achieved	✓	✓	
Jump height relative to dog height			
Approach to competition planning			
Advance competition planning	✓		
Trial weekends per year	✓		
Average runs per trial day	✓	✓	
Average days per trial weekend	✓		
Grass surface	✓		
Dirt surface	✓	✓	✓
Sand surface			
Artificial turf surface	✓	✓	✓
Foam surface			
Rubber mat surface	✓	✓	
Other surface			
**Training factors**			
Age started any agility training	✓	✓	
Age of first fun match	✓		
Age at first trial	✓		
Age started any jump training	✓	✓	
Age started elbow height jumps	✓		
Age started full height jumps			
Age started backside jump training	✓		
Age started backside at full height			
Age starting any tunnel training	✓		
Age started curved tunnel training	✓	✓	
Age started Aframe training	✓		
Age started dogwalk training	✓	✓	
Age started teeter training	✓		
Age started any weave training	✓		
Age started sequencing with closed weaves	✓	✓	
Aframe contact behavior	✓	✓	
Dogwalk contact behavior	✓		
Teeter contact behavior	✓		
Weave training method	✓	✓	✓

*Variables moved from step 1 to step 2 if they were significant (p < 0.2) in models adjusted for age of the dog. In step 2, backward selection was conducted within each block of variables until all variables in the block were significant (p < 0.2). Final model building (step 3) was done via backward selection starting with all remaining variables after the model building in step 2*.

**Table 2:** Coefficients from final adjusted model of risk factors of iliopsoas injury.

	**Adjusted OR**	**Adjusted**
	**(95% CI)**	***p*-value**
Dog age (per 1 year older)	1.13 (1.09, 1.18)	<0.001
**Breed**		<0.001
Border collie	1.99 (1.51, 2.63)	
Mixed breed	0.94 (0.60, 1.46)	
Shetland sheepdog	1.32 (0.83, 2.10)	
Australian shepherd	1.51 (0.97, 2.36)	
Other	REFERENCE	
**Acquired w/agility in mind**		0.001
No	0.57 (0.41, 0.78)	
Yes	REFERENCE	
**Handler medical training/experience**		0.022
None of these	REFERENCE	
Veterinarian	0.98 (0.52, 1.86)	
Licensed vet tech	0.62 (0.26, 1.45)	
Veterinary assistant	2.51 (1.39, 4.53)	
Human health care professional	1.17 (0.84, 1.63)	
**Dirt surface**		0.017
Never competed	REFERENCE	
<6 times per year	1.53 (1.13, 2.06)	
6+ times per year	1.46 (1.04, 2.06)	
**Artificial turf surface**		<0.001
Never competed	REFERENCE	
<6 times per year	1.07 (0.77, 1.49)	
6+ times per year	1.77 (1.32, 2.37)	
**Weave training method**		0.002
2 × 2	REFERENCE	
Channel	0.59 (0.44, 0.79)	
Guide wires	0.79 (0.53, 1.17)	
Other	0.67 (0.45, 1.00)	

## Discussion

This study found several factors associated with increased risk of developing an iliopsoas injury in dogs competing in agility. Border Collies had increased odds of injury, which aligns with previously published data (1, 12, 14). This consistent finding may be related to the breed's high drive and athleticism, which tends to be one of the primary reasons they are chosen for agility. In human athletes, high speed, intense acceleration, and the tendency to override pain and keep performing, puts athletes at increased risk for muscle injury (15). It is possible that these same characteristics, common in the Border Collie breed, may increase their risk of injury. Speed has been postulated to be a cause for increased injury risk in racing greyhounds as well (16).

Even after adjusting for breed, dogs had a decreased risk for iliopsoas injury if they were not acquired specifically for participation in agility competition, compared to those dogs acquired with the intent to participate in agility. This association may, once again, reflect the greater injury risk among dogs of breeds with higher drive, faster speed, and greater athleticism that are sought by handlers acquiring a dog specifically for agility. It may also be due to handlers who have acquired a dog specifically for agility being more proactive in seeking veterinary care for injury. It is possible that handlers of dogs who were acquired specifically for agility may be more astute in detecting minor changes in their dog's gait or performance, leading to more frequent suspicion and diagnosis of iliopsoas injury. A similar finding was suggested by the Spinella et al. study, where Border Collie owners, and those that participated in agility, sought veterinary care sooner after injury than the other breeds represented (9). While one might assume that dogs acquired with the intent to participate in agility may have increased intensity of training and competition, thereby increasing injury risk, other variables associated with frequency of training and competition did not appear to increase risk of iliopsoas injury in this survey.

Counter to our original hypotheses, only one training factor, the weave obstacle training method, was associated with iliopsoas injury in the final model. There are a variety of methods for training dogs to weave through the weave poles, but in this study the 2 × 2 weave training method was associated with increased iliopsoas injury risk compared to other methods. It is unknown why the 2 × 2 weave training method was associated with increased risk of iliopsoas injury. A recent study described the types of gait patterns dogs use while performing the weave obstacle, but the biomechanical effects on the body have not been evaluated (17). It is unknown if the weave training methods influence the preferred gait pattern through the weave poles, or how the training methods differ in biomechanical effect on the body. It is possible that the 2 × 2 training method requires greater repetitions, or has increased forces on the body, thereby increasing the risk of a repetitive stress iliopsoas injury. It is also possible that the 2 × 2 training method is not directly correlated with iliopsoas injury, but that the dogs of handlers who are choosing the 2 × 2 training method are at increased injury risk due to other influences not evaluated in this survey. Anecdotally, age of initiation of training on various obstacles is frequently thought to be related to risk for injury. However, age of starting various obstacle training was not associated with risk of iliopsoas injury in the final model. Unlike many of the demographic variables, most of the training factors are potentially modifiable, so further evaluation in prospective studies is warranted.

Based on this survey data, competition factors did not influence injury risk as much as initially hypothesized. The number of trial weekends, days of competition per weekend, and number of runs per competition day were not significantly associated with an increased risk of iliopsoas injury, indicating that competition schedule alone may not significantly contribute to iliopsoas injury risk. In the human sports medicine literature, competition frequency and number of games played are only two of many variables that influence total workload for an athlete, which has been shown to be directly related to risk of musculoskeletal injury (18–20). In addition to session frequency, factors such as distance, duration, repetitions, power output, heart rate, and exertion all contribute to external and internal load measures (18–20). It is also established that there are other variables such as psychological stresses, travel, level of fitness, as well as metabolic, hormonal and genetic factors that all contribute to overall load (18–20). A better understanding of competition factors and physiologic variables that contribute to a canine athlete's workload is necessary to determine the relationship with musculoskeletal injury risk.

Of the competition variables, competition surface was associated with risk of iliopsoas injury. Canine athletes competing more frequently on dirt or artificial turf were more likely to have experienced an iliopsoas injury. Anecdotally, many agility competitors prefer dirt or artificial turf due to perceived lower risk of injury from slipping. However, artificial turf varies widely in composition, which can affect the surface properties and traction creating documented alterations in ankle and knee kinematics and kinetics in human athletes (21). Composition and quality of dirt, as well as maintenance of the surface during competitions, also vary widely. Evaluation of surfaces and their impacts on hind limb kinematics has been explored in racing greyhounds, but has not been specifically explored in agility dogs or in relation to injury risk (22). The association between iliopsoas injury risk and surface may be related to the footing itself, or may be correlated with higher speeds in these competition settings. It should be noted that this finding is specific to competition surface and does not account for running surfaces used during training. It is possible that athletes have a higher level of intensity or speed during competition, making the impacts of surface more significant, but this possibility cannot be evaluated with the data from this survey.

Jumping has frequently been suggested as a possible cause of injury for agility athletes, and hesitancy to jump is often one of the first symptoms described after an iliopsoas injury (23–28). Iliopsoas injury has been postulated to result from microtrauma from repetitive jumping, but jumping frequency (based on number of runs per day), age at which jump training was started, and the heights of jumps were not associated with odds of iliopsoas injury in this study. The evaluation the biomechanics of the iliopsoas muscle during agility competition and training activities may reveal additional information about iliopsoas function and injury.

It should be noted that handlers reported that a non-veterinary professional was primarily responsible for treatment decisions in a large percentage of cases. This may reflect increased access to these professionals by the agility community, as well as responsiveness of these professionals to injury concerns. It is important for veterinary professionals to recognize the frequency at which sports medicine treatment decisions (and likely also diagnoses) are being made by other caregivers, as this highlights a potential lack of veterinary involvement in the early stages of injury. This disparity also presents an opportunity for education and growth within veterinary practice to better serve this subset of patients.

While this survey was unable to evaluate precise treatment protocols for iliopsoas injuries due to the lack of acquisition of veterinary medical records, owner-reported treatment for dogs with iliopsoas injury was consistent with current recommendations including rest, pharmaceutical management, and rehabilitation (8, 29). In this survey, many handlers reported using formal rehabilitation therapy and/or in-home rehabilitation as part of their dog's treatment protocol. Rehabilitation is inherently diverse, not only across patients and conditions, but also across practitioners. While we can say that most of the handlers sought rehabilitation as part of their therapeutic plan, details on modalities, duration, frequency, and benefit were not included in this survey. With iliopsoas injury being a common injury reported in the agility population, further evaluation of the effectiveness of rehabilitation techniques, timing, and duration are needed to develop the most appropriate therapeutic plans for patients with the diagnosis of iliopsoas injury.

Some information on recurrence of iliopsoas injury, recovery from injury and return to agility can be inferred from this survey, though not without significant limitations. In this study, 36% of dogs reported a history of multiple iliopsoas strains. Once a muscle or tendon is injured it is more prone to repeat injury or chronic conditions secondary to long-term repetitive overuse/guarding, intermittent inflammation, and repeat micro-injury (5, 15, 30). One study evaluated musculoskeletal ultrasound in agility dogs with iliopsoas injury and reported evidence of both acute and chronic inflammation within the same patient in 62.8% of cases, consistent with repeat micro-injury (5). With regards to iliopsoas injury recovery, this survey found that 56% of dogs were able to return to competition within 3 months, consistent with a median of 91 days to full recovery reported by Spinella et al. (9). The remainder of dogs had a more prolonged convalescence, with 24% returning in 4–6 months, 14% being out of agility for longer than 6 months, and 6% officially retiring. Iliopsoas injuries can be primary, or can occur secondary to orthopedic and neurologic conditions. Underlying orthopedic or neurologic conditions can cause a change in the gait patterning in order to protect the affected joint or region, often by limiting range of motion and relying heavily on muscles like the iliopsoas for stability and compensation (5). It is possible that dogs with secondary iliopsoas injuries could have contributed to the cases with longer recovery times due to the effect of the underlying condition. The nuances of both acute and chronic iliopsoas injury, existence of comorbidities, degree of severity and tendon involvement, and variety in management approaches, make predicting an athlete's ability to return to sport challenging and warrants further exploration.

The results of this study should be interpreted with the understanding that there are significant inherent limitations in a retrospective, owner/handler reported survey, including difficulty in injury recall, self-selection bias, and lack of confirmatory veterinary diagnosis. Participant recall may affect survey outcomes, however, self-reporting and parental reporting in humans has shown good accuracy, especially for major injuries (31–33). It has been established that those who self-select for a survey when it evaluates a topic they care about personally, tend to provide more complete and higher quality data when compared to randomly selected participants, potentially minimizing self-selection bias (34). Agility dog handlers demonstrate a high interest level and commitment to the health of their dogs, as indicated by the 4,197 respondents to this survey, which represents the largest participation in this type of study to date. One of the most substantial limitations of this survey is the lack of access to veterinary records and diagnostics performed. Without the veterinary records it is unknown how the iliopsoas injury was diagnosed, and whether a definitive diagnosis was made. Diagnosis of iliopsoas injuries can be challenging, and presumptive diagnosis is often based on physical examination alone. It is unknown how many of the reported cases had advanced imaging, such as musculoskeletal ultrasound or magnetic resonance imaging (MRI) for confirmation, versus presumptive, and possible inaccurate, diagnosis. Another limitation of these data is the ability to assess certain outcomes due to confounding factors. Some outcomes, such as time to return to competition by those treated by veterinarians / veterinary specialists vs. non-veterinarians, are likely heavily confounded by injury severity (e.g., dogs with more significant injuries were more likely to be treated by a veterinary professional). Focused, prospective studies would allow for improved characterization of iliopsoas injuries and resolution of many of the limitations inherent in this survey.

In conclusion, this survey provides insight into possible risk factors associated with iliopsoas injuries, but also indicates a significant need for studies on pathophysiology of iliopsoas injuries in sporting dogs, as well as best treatment strategies. Further exploration into the relationship of iliopsoas injuries and common comorbidities, the impact of footing on kinematics and injuries in agility courses, as well as weave pole training techniques, is warranted based on these results to help improve the safety of agility as a sport and also better manage one of the most commonly reported injuries. Some of the final risk factors cannot be modified (breed, intended use and handler profession), but can be taken into consideration for injury prevention strategies.

## Data Availability Statement

The raw data supporting the conclusions of this article will be made available by the authors, without undue reservation.

## Ethics Statement

Ethical review and approval was not required for the study on human participants in accordance with the local legislation and institutional requirements. The patients/participants provided their written informed consent to participate in this study. Ethical review and approval was not required for the animal study because The Ohio State University Office of Responsible Research Practices determined the project was exempt from IRB review because it was an owner-based internet survey and the information was recorded without direct or indirect identifiers. Written informed consent was obtained from the owners for the participation of their animals in this study.

## Author Contributions

LF and JR participated in data evaluation and writing the manuscript. AP and NK assisted in study design, data collection, data evaluation, and writing the manuscript. AS participated in study design, statistical analysis, and writing the manuscript. All authors contributed to the article and approved the submitted version.

## Conflict of Interest

The authors declare that the research was conducted in the absence of any commercial or financial relationships that could be construed as a potential conflict of interest.

## Publisher's Note

All claims expressed in this article are solely those of the authors and do not necessarily represent those of their affiliated organizations, or those of the publisher, the editors and the reviewers. Any product that may be evaluated in this article, or claim that may be made by its manufacturer, is not guaranteed or endorsed by the publisher.
