# Supplementary material for: Internet Survey Evaluation of Iliopsoas Injury in Dogs Participating in Agility Competitions
Source: Front Vet Sci. 2022 Jul 8;9:930450. doi: 10.3389/fvets.2022.930450 (PMC9305456; doi:10.3389/fvets.2022.930450)
Supplement: Supplementary file 1 [file Table_1.docx]

**Supplemental Tables**

Supplemental Table 1. Age adjusted associations between demographic risk factors and iliopsoas injury history.

|  | N (%) | Age adjusted OR (95% CI) | Age adjusted  p-value |
| --- | --- | --- | --- |
| **Dog demographics** |  |  |  |
| Height & Weight together |  |  | 0.027 ^a^ |
| Dog Height (per 4 inches taller) | n/a | 1.27 (1.01, 1.56) |  |
| Dog Weight (per 10 pounds heavier) | n/a | 0.94 (0.83, 1.07) |  |
| Breed |  |  | <0.001^a^ |
| Border Collie | 934 (22.3) | 2.24 (1.72, 2.93) |  |
| Mixed Breed | 555 (13.2) | 0.77 (0.51, 1.19) |  |
| Shetland Sheepdog | 277 (6.6) | 1.54 (0.98, 2.42) |  |
| Australian Shepherd | 285 (6.8) | 1.65 (1.06, 2.55) |  |
| Other | 2146 (51.1) | REFERENCE |  |
| Country/Region |  |  | 0.001^a^ |
| United States | 2570 (61.2) | REFERENCE |  |
| UK / Ireland | 469 (11.2) | 0.50 (0.31, 0.80) |  |
| Canada | 392 (9.3) | 0.82 (0.55, 1.22) |  |
| Cont. Europe | 343 (8.2) | 0.37 (0.20, 0.68) |  |
| Australia | 163 (3.9) | 0.77 (0.41, 1.44) |  |
| Other | 260 (6.2) | 1.20 (0.78, 1.86) |  |
| Age brought dog home |  |  | 0.19^a^ |
| <8 weeks | 868 (20.7) | 0.94 (0.70, 1.27) |  |
| 8-12 weeks | 2246 (53.6) | REFERENCE |  |
| 13-15 weeks | 230 (5.5) | 1.59 (1.04, 2.44) |  |
| 4-6 months | 241 (5.8) | 0.81 (0.47, 1.38) |  |
| 7-12 months | 238 (5.7) | 0.91 (0.55, 1.52) |  |
| >12 months | 366 (8.7) | 0.79 (0.51, 1.23) |  |
| How acquired |  |  | 0.045^a^ |
| Breeder | 3092 (73.8) | REFERENCE |  |
| Rescue / Shelter | 708 (16.9) | 0.71 (0.46, 1.09) |  |
| Other | 389 (9.3) | 0.70 (0.50, 0.97) |  |
| Acquired w/agility in mind |  |  | <0.001^a^ |
| No | 1214 (29.0) | 0.42 (0.31, 0.56) |  |
| Yes | 2978 (71.0) | REFERENCE |  |
| Agility main sport focus |  |  | 0.024^a^ |
| Yes | 3016 (71.9) | REFERENCE |  |
| Mostly | 824 (19.6) | 1.02 (0.77, 1.36) |  |
| No | 356 (8.5) | 0.47 (0.27, 0.81) |  |
| Sex / Neuter status |  |  | 0.89 |
| Male, Intact | 671 (16.9) | REFERENCE |  |
| Female, Intact | 486 (12.2) | 1.01 (0.62, 1.63) |  |
| Male, Neutered <10 months | 377 (9.5) | 0.84 (0.51, 1.40) |  |
| Male, Neutered 10-18 months | 538 (13.5) | 1.07 (0.69, 1.66) |  |
| Male, Neutered >24 months | 421 (10.6) | 0.94 (0.58, 1.52) |  |
| Female, Spayed <1 cycle | 539 (13.6) | 1.12 (0.73, 1.72) |  |
| Female, Spayed 1 cycle | 367 (9.2) | 1.16 (0.72, 1.88) |  |
| Female, Spayed >1 cycle | 580 (14.6) | 1.19 (0.78, 1.81) |  |
| Front dew claws |  |  | 0.59 |
| Intact | 2951 (70.3) | REFERENCE |  |
| Removed / Unknown | 1240 (29.7) | 0.93 (0.73, 1.20) |  |
| Rear dew claws |  |  | 0.22 |
| Intact | 782 (18.6) | 0.82 (0.60, 1.12) |  |
| Removed or born without / Unknown | 3415 (81.4) | REFERENCE |  |
| Docked tail |  |  | 0.90 |
| Yes | 760 (18.1) | 0.98 (0.73, 1.32) |  |
| No / unknown | 3435 (81.9) | REFERENCE |  |
| Growth plate x-rays |  |  | <0.001^a^ |
| Not done | 3432 (81.8) | REFERENCE |  |
| Done at least once | 763 (18.2) | 1.67 (1.28, 2.18) |  |
| **Handler demographics** |  |  |  |
| Handler current age |  |  | 0.61 |
| 18-24 | 208 (5.0) | REFERENCE |  |
| 25-34 | 657 (15.7) | 1.62 (0.81, 3.26) |  |
| 35-44 | 634 (15.2) | 1.45 (0.72, 2.93) |  |
| 45-54 | 866 (20.8) | 1.41 (0.71, 2.81) |  |
| 55-64 | 1176 (28.2) | 1.59 (0.81, 3.12) |  |
| 65+ | 633 (15.2) | 1.25 (0.62, 2.54) |  |
| Handler gender |  |  | 0.087^a^ |
| Female | 3915 (93.8) | REFERENCE |  |
| Male / Other gender identity | 212 (5.1) | 0.63 (0.37, 1.07) |  |
| Handler education |  |  | 0.27 |
| Graduate or professional degree | 1389 (33.5) | REFERENCE |  |
| 4-year college | 1296 (31.2) | 0.94 (0.71, 1.24) |  |
| 2-year college | 452 (10.9) | 1.10 (0.75, 1.60) |  |
| Some college | 586 (14.1) | 0.70 (0.47, 1.03) |  |
| HS degree (or less) | 425 (10.3) | 0.77 (0.50, 1.19) |  |
| Handler profession |  |  | 0.070 ^a^ |
| Not a dog trainer | 2738 (66.1) | REFERENCE |  |
| Paid trainer, not primary job | 1054 (25.4) | 1.11 (0.85, 1.45) |  |
| Professional trainer | 352 (8.5) | 1.54 (1.06, 2.24) |  |
| Handler medical training / experience |  |  | 0.028^a^ |
| None of these | 3215 (77.9) | REFERENCE |  |
| Veterinarian | 149 (3.6) | 1.04 (0.55, 1.96) |  |
| Licensed vet tech | 106 (2.6) | 0.74 (0.32, 1.72) |  |
| Veterinary assistant | 96 (2.3) | 2.45 (1.39, 4.34) |  |
| Human health care professional | 562 (13.6) | 1.20 (0.87, 1.67) |  |
| Handler agility experience |  |  | 0.016^a^ |
| <3 years | 410 (9.8) | 0.38 (0.20, 0.73) |  |
| 3-5 years | 722 (17.2) | 0.82 (0.57, 1.17) |  |
| 6-10 years | 1054 (25.2) | 0.88 (0.65, 1.18) |  |
| 11-15 years | 696 (16.6) | 1.15 (0.84, 1.58) |  |
| >15 years | 1308 (31.2) | REFERENCE |  |
| Handler competed at national level |  |  | 0.015^a^ |
| No | 1893 (45.2) | REFERENCE |  |
| Yes | 2299 (54.8) | 1.34 (1.06, 1.71) |  |
| Handler competed at international level |  |  | 0.39 |
| No | 3744 (89.5) | REFERENCE |  |
| Yes | 439 (10.5) | 0.84 (0.56, 1.25) |  |

^a^p<0.20 and retained for model building

Supplemental Table 2. Age adjusted associations between competition risk factors and iliopsoas injury history.

|  | N (%) | Age adjusted OR (95% CI) | Age adjusted  p-value |
| --- | --- | --- | --- |
| Primary organization |  |  | 0.043^a^ |
| AKC | 1172 (27.9) | REFERENCE |  |
| CPE | 344 (8.2) | 0.78 (0.50, 1.22) |  |
| USDAA | 296 (7.1) | 1.12 (0.74, 1.72) |  |
| NADAC | 112 (2.7) | 1.17 (0.62, 2.21) |  |
| AAC (Canada) | 225 (5.4) | 0.64 (0.36, 1.14) |  |
| Other North American | 813 (19.4) | 0.86 (0.62, 1.19) |  |
| FCI agility | 756 (18.0) | 0.57 (0.39, 0.83) |  |
| Other non-North American | 477 (11.4) | 0.66 (0.43, 1.02) |  |
| Highest level achieved |  |  | <0.001^a^ |
| Entry level | 596 (14.2) | 0.39 (0.24, 0.65) |  |
| Intermediate level | 766 (18.3) | 0.67 (0.47, 0.94) |  |
| High level | 2829 (67.5) | REFERENCE |  |
| Jump height difference |  |  | 0.39 |
| Jumping >4” above height | 144 (3.5) | 0.46 (0.20, 1.07) |  |
| Jumping 2-4” above height | 299 (7.3) | 0.98 (0.62, 1.57) |  |
| Jumping 0-2” above height | 853 (20.9) | 0.86 (0.61, 1.19) |  |
| Jumping 0-2” below height | 1158 (28.4) | REFERENCE |  |
| Jumping 2-4” below height | 797 (19.6) | 0.85 (0.61, 1.18) |  |
| Jumping 4-6” below height | 485 (11.9) | 0.74 (0.50, 1.10) |  |
| Jumping >6” below height | 339 (8.3) | 0.70 (0.45, 1.11) |  |
| Approach to competition planning |  |  | 0.43 |
| Plan around availability / schedule | 2801 (67.0) | REFERENCE |  |
| Plan around a big event | 101 (2.4) | 1.45 (0.74, 2.84) |  |
| Mix of the two | 1107 (26.5) | 1.18 (0.91, 1.53) |  |
| Other approach | 171 (4.1) | 0.90 (0.49, 1.64) |  |
| Advance competition planning |  |  | 0.059 ^a^ |
| 1-2 months | 1533 (36.7) | REFERENCE |  |
| 3-6 months | 1910 (45.7) | 1.33 (1.02, 1.73) |  |
| 6-12 months | 631 (15.1) | 1.42 (1.01, 2.00) |  |
| 12+ months | 104 (2.5) | 0.69 (0.27, 1.74) |  |
| Trial weekends per year |  |  | 0.024 ^a^ |
| <5 weekends | 448 (10.7) | 0.46 (0.24, 0.86) |  |
| 5-10 weekends | 918 (21.9) | 0.84 (0.52, 1.37) |  |
| 11-15 weekends | 1082 (25.8) | 1.03 (0.65, 1.64) |  |
| 16-20 weekends | 906 (21.6) | 1.15 (0.72, 1.83) |  |
| 21-25 weekends | 530 (12.7) | 1.04 (0.63, 1.72) |  |
| 26+ weekends | 304 (7.3) | REFERENCE |  |
| Average runs per trial day |  |  | 0.14 ^a^ |
| 1-2 runs per day | 1067 (25.5) | REFERENCE |  |
| 3-4 runs per day | 2444 (58.4) | 1.30 (0.98, 1.72) |  |
| 5+ runs per day | 677 (16.2) | 1.06 (0.73, 1.55) |  |
| Average days per trial weekend |  |  | 0.18 ^a^ |
| Only 1 day | 485 (11.6) | 0.68 (0.45, 1.04) |  |
| 1 or 2 days; it depends | 1680 (40.1) | 0.80 (0.62, 1.03) |  |
| Usually 2 days, sometimes 3 | 1701 (40.6) | REFERENCE |  |
| As many as possible (often 3) | 320 (7.6) | 0.92 (0.60, 1.41) |  |
| Grass surface |  |  | 0.18 ^a^ |
| Never competed | 767 (18.3) | REFERENCE |  |
| <6 times per year | 1882 (45.0) | 1.11 (0.80, 1.53) |  |
| 6+ times per year | 1536 (36.7) | 0.87 (0.62, 1.22) |  |
| Dirt surface |  |  | <0.001^a^ |
| Never competed | 1597 (38.2) | REFERENCE |  |
| <6 times per year | 1698 (40.6) | 1.88 (1.42, 2.49) |  |
| 6+ times per year | 890 (21.3) | 1.70 (1.23, 2.35) |  |
| Sand surface |  |  | 0.47 |
| Never competed | 2645 (63.2) | REFERENCE |  |
| <6 times per year | 1253 (29.9) | 1.05 (0.82, 1.34) |  |
| 6+ times per year | 287 (6.9) | 0.74 (0.44, 1.26) |  |
| Turf surface |  |  | <0.001^a^ |
| Never competed | 1660 (39.7) | REFERENCE |  |
| <6 times per year | 1095 (26.2) | 1.30 (0.95, 1.78) |  |
| 6+ times per year | 1430 (34.2) | 2.03 (1.55, 2.67) |  |
| Foam surface |  |  | 0.94 |
| Never competed | 3522 (84.2) | REFERENCE |  |
| <6 times per year | 494 (11.8) | 1.06 (0.75, 1.49) |  |
| 6+ times per year | 169 (4.0) | 1.05 (0.59, 1.84) |  |
| Rubber surface |  |  | 0.034 ^a^ |
| Never competed | 2761 (66.0) | REFERENCE |  |
| <6 times per year | 1054 (25.2) | 1.25 (0.97, 1.60) |  |
| 6+ times per year | 370 (8.8) | 0.68 (0.43, 1.09) |  |
| Other surface |  |  | 0.80 |
| Never competed | 3972 (94.9) | REFERENCE |  |
| <6 times per year | 141 (3.4) | 0.88 (0.46, 1.70) |  |
| 6+ times per year | 72 (1.7) | 0.75 (0.27, 2.07) |  |

^a^p<0.20 and retained for model building

Supplemental Table 3. Age adjusted associations between training risk factors and iliopsoas injury history.

|  | N (%) | Age adjusted OR (95% CI) | Age adjusted  p-value |
| --- | --- | --- | --- |
| First started any agility-specific training |  |  | 0.002^a^ |
| < 16 weeks | 625 (14.9) | 1.47 (0.90, 2.38) |  |
| 4-6 months | 876 (20.9) | 2.20 (1.42, 3.39) |  |
| 6-12 months | 1211 (28.9) | 1.91 (1.25, 2.90) |  |
| 13-18 months | 657 (15.7) | 1.22 (0.75, 1.98) |  |
| 19-24 months | 295 (7.0) | 1.44 (0.81, 2.54) |  |
| 2+ years | 533 (12.7) | REFERENCE |  |
| Age competed in first fun match |  |  | 0.039^a^ |
| <12 months | 86 (2.1) | 1.71 (0.72, 4.07) |  |
| 12-15 months | 566 (13.6) | 1.85 (1.14, 2.99) |  |
| 16-18 months | 951 (22.8) | 1.83 (1.18, 2.85) |  |
| 19-24 months | 899 (21.6) | 1.27 (0.80, 2.02) |  |
| 25-30 months | 320 (7.7) | 1.57 (0.91, 2.71) |  |
| 31-36 months | 106 (2.5) | 0.64 (0.22, 1.86) |  |
| 3+ years | 455 (10.9) | REFERENCE |  |
| N/A – no fun match | 787 (18.9) | 1.29 (0.81, 2.06) |  |
| Age competed in first trial |  |  | 0.012^a^ |
| <16 months | 203 (4.9) | 1.22 (0.63, 2.37) |  |
| 16-18 months | 837 (20.0) | 2.06 (1.41, 3.03) |  |
| 19-24 months | 1566 (37.5) | 1.48 (1.03, 2.12) |  |
| 25-30 months | 640 (15.3) | 1.60 (1.05, 2.43) |  |
| 31-36 months | 200 (4.8) | 1.52 (0.84, 2.77) |  |
| 3+ years old | 732 (17.5) | REFERENCE |  |
| Age any jumps |  |  | 0.008^a^ |
| > 18 months | 701 (16.9) | REFERENCE |  |
| < 3 months | 101 (2.4) | 0.80 (0.28, 2.30) |  |
| 3-6 months | 500 (12.1) | 1.56 (0.98, 2.50) |  |
| 7-9 months | 852 (20.6) | 1.55 (1.03, 2.35) |  |
| 10-12 months | 1015 (24.5) | 2.12 (1.45, 3.11) |  |
| 13-15 months | 744 (18.0) | 1.63 (1.07, 2.47) |  |
| 16-18 months | 233 (5.6) | 1.52 (0.85, 2.71) |  |
| Age elbow height jumps |  |  | 0.021^a^ |
| > 18 months | 885 (21.7) | REFERENCE |  |
| < 7 months | 72 (1.8) | 0.81 (0.25, 2.67) |  |
| 7-9 months | 281 (6.9) | 1.45 (0.85, 2.46) |  |
| 10-12 months | 897 (22.0) | 1.62 (1.12, 2.35) |  |
| 13-15 months | 1385 (34.0) | 1.81 (1.29, 2.53) |  |
| 16-18 months | 553 (13.6) | 1.50 (0.98, 2.28) |  |
| Age full height jumps |  |  | 0.90 |
| > 18 months | 1461 (35.6) | REFERENCE |  |
| < 10 months | 54 (1.3) | 1.37 (0.67, 2.80) |  |
| 10-12 months | 349 (8.5) | 1.13 (0.73, 1.76) |  |
| 13-15 months | 1139 (27.7) | 1.09 (0.77, 1.53) |  |
| 16-18 months | 1104 (26.9) | 1.05 (0.75, 1.47) |  |
| Age backside at any height |  |  | 0.15^a^ |
| > 18 months | 1907 (50.5) | REFERENCE |  |
| < 10 months | 96 (2.5) | 1.22 (0.65, 2.28) |  |
| 10-12 months | 354 (9.4) | 1.14 (0.79, 1.65) |  |
| 13-15 months | 701 (18.6) | 1.38 (1.06, 1.81) |  |
| 16-18 months | 718 (19.0) | 1.26 (0.96, 1.64) |  |
| Age backside at full height |  |  | 0.49 |
| > 18 months | 2246 (58.9) | REFERENCE |  |
| < 13 months | 176 (4.6) | 1.20 (0.68, 2.13) |  |
| 13-15 months | 579 (15.2) | 1.03 (0.71, 1.49) |  |
| 16-18 months | 810 (21.3) | 1.26 (0.93, 1.69) |  |
| Tunnel age |  |  | 0.001^a^ |
| > 18 months | 641 (15.5) | REFERENCE |  |
| < 3 months | 600 (14.5) | 1.07 (0.64, 1.77) |  |
| 3-6 months | 1080 (26.2) | 2.24 (1.50, 3.35) |  |
| 7-9 months | 713 (17.3) | 1.99 (1.29, 3.06) |  |
| 10-12 months | 513 (12.4) | 1.67 (1.04, 2.66) |  |
| 13-15 months | 396 (9.6) | 1.49 (0.90, 2.47) |  |
| 16-18 months | 184 (4.5) | 1.77 (0.94, 3.33) |  |
| Curved tunnel age |  |  | <0.001^a^ |
| > 18 months | 705 (17.0) | REFERENCE |  |
| < 3 months | 208 (5.0) | 0.80 (0.37, 1.75) |  |
| 3-6 months | 857 (20.6) | 1.71 (1.13, 2.57) |  |
| 7-9 months | 935 (22.5) | 2.34 (1.59, 3.45) |  |
| 10-12 months | 684 (16.5) | 1.51 (0.98, 2.34) |  |
| 13-15 months | 506 (12.2) | 1.70 (1.08, 2.66) |  |
| 16-18 months | 259 (6.2) | 1.26 (0.69, 2.30) |  |
| Aframe Age |  |  | 0.014^a^ |
| > 18 months | 1159 (28.1) | REFERENCE |  |
| < 10 months | 317 (7.7) | 1.27 (0.77, 2.09) |  |
| 10-12 months | 821 (19.9) | 1.70 (1.21, 2.39) |  |
| 13-15 months | 1203 (29.2) | 1.66 (1.20, 2.28) |  |
| 16-18 months | 626 (15.2) | 1.49 (1.02, 2.19) |  |
| Dogwalk Age |  |  | 0.004^a^ |
| > 18 months | 1150 (27.9) | REFERENCE |  |
| < 10 months | 423 (10.3) | 1.59 (1.04, 2.43) |  |
| 10-12 months | 934 (22.6) | 1.65 (1.18, 2.32) |  |
| 13-15 months | 1083 (26.2) | 1.83 (1.33, 2.53) |  |
| 16-18 months | 537 (13.0) | 1.29 (0.85, 1.96) |  |
| Teeter Age |  |  | 0.037^a^ |
| > 18 months | 1134 (28.9) | REFERENCE |  |
| < 10 months | 389 (9.9) | 1.55 (1.02, 2.36) |  |
| 10-12 months | 847 (21.6) | 1.66 (1.19, 2.31) |  |
| 13-15 months | 1010 (25.8) | 1.36 (0.97, 1.89) |  |
| 16-18 months | 543 (13.8) | 1.20 (0.79, 1.80) |  |
| Any weaves age |  |  | 0.013^a^ |
| > 18 months | 1037 (24.8) | REFERENCE |  |
| < 7 months | 205 (4.9) | 0.97 (0.50, 1.87) |  |
| 7-9 months | 462 (11.1) | 1.29 (0.83, 1.99) |  |
| 10-12 months | 825 (19.7) | 1.35 (0.94, 1.94) |  |
| 13-15 months | 1148 (27.5) | 1.79 (1.30, 2.47) |  |
| 16-18 months | 503 (12.0) | 1.33 (0.87, 2.01) |  |
| Sequences closed weaves |  |  | 0.001^a^ |
| > 18 months | 1464 (35.0) | REFERENCE |  |
| < 10 months | 135 (3.2) | 0.96 (0.43, 2.13) |  |
| 10-12 months | 495 (11.8) | 1.44 (0.97, 2.14) |  |
| 13-15 months | 1250 (29.9) | 1.74 (1.30, 2.34) |  |
| 16-18 months | 838 (20.0) | 1.86 (1.35, 2.56) |  |
| Aframe contact |  |  | 0.050^a^ |
| 2 on 2 off | 1900 (47.7) | REFERENCE |  |
| 4 on | 133 (3.3) | 0.25 (0.08, 0.79) |  |
| Other / no specific behavior | 164 (4.1) | 0.90 (0.50, 1.63) |  |
| Running | 1785 (44.8) | 0.81 (0.63, 1.03) |  |
| Dogwalk contact |  |  | 0.015^a^ |
| 2 on 2 off | 2528 (60.3) | REFERENCE |  |
| 4 on | 199 (4.8) | 0.42 (0.21, 0.87) |  |
| Other / no specific behavior | 146 (3.5) | 0.97 (0.54, 1.75) |  |
| Running | 1317 (31.4) | 0.72 (0.55, 0.94) |  |
| Teeter contact |  |  | 0.068^a^ |
| 2 on 2 off | 2203 (52.7) | REFERENCE |  |
| 4 on (down) | 311 (7.4) | 0.87 (0.56, 1.37) |  |
| 4 on (standing) | 1200 (28.7) | 0.74 (0.56, 0.97) |  |
| No specific behavior | 240 (5.7) | 0.52 (0.29, 0.95) |  |
| Other | 224 (5.4) | 0.71 (0.41, 1.23) |  |
| Weave training method |  |  | 0.001^a^ |
| 2 x 2 | 1912 (45.8) | REFERENCE |  |
| Channel | 1329 (31.8) | 0.58 (0.44, 0.77) |  |
| Guide wires | 462 (11.1) | 0.68 (0.46, 1.01) |  |
| Other | 474 (11.4) | 0.67 (0.45, 0.98) |  |

^a^p<0.20 and retained for model building
